# Multiomics profiles of genome-wide alterations in H3K27ac in different lung lobes after acute graft-*versus*-host disease with MSCs treatment

**DOI:** 10.3389/fimmu.2025.1570916

**Published:** 2025-05-15

**Authors:** Zixuan Lu, Yuming Zhou, Chengyu Li, A. M. Abd El-Aty, Chengxia Liu, Xiying Luan, Bin Wang, Guoyan Wang

**Affiliations:** ^1^ Department of Immunology, Binzhou Medical University, Yantai, China; ^2^ Medical Laboratory of the Second School of Clinical Medicine, Binzhou Medical University, Yantai, China; ^3^ Department of Pharmacology, Faculty of Veterinary Medicine, Cairo University, Giza, Egypt; ^4^ Department of Medical Pharmacology, Medical Faculty, Ataturk University, Erzurum, Türkiye; ^5^ Department of Gastroenterology and Institute of Digestive Disease, Binzhou Medical University Hospital, Binzhou, Shandong, China

**Keywords:** acute graft-versus-host disease, human placenta-derived mesenchymal stem cells, different lung lobes, H3K27ac, epigenetic regulation

## Abstract

The molecular characteristics of acute graft-*versus*-host disease (aGVHD) in different lung lobes and the treatment of aGVHD with mesenchymal stem cells are still poorly understood. In addition, despite the important role of acetylation on lysine 27 of histone H3 (H3K27ac) in the inflammatory response, little is known about genome-wide H3K27ac in GVHD and MSC treatment. In this study, we described 55 paired transcriptomes and genome-wide H3K27ac in five lung lobes, with groups designated as follows: control, GVHD, human placenta-derived MSC (hPMSC)-treated, and PBS-treated groups. We observed that inflammatory pathways were upregulated in GVHD but downregulated in hPMSCs. One algorithm was designed to identify the genes implicated in the prevention of GVHD by hPMSCs (the Rein02 gene), shedding light on a gene set with 892 Rein02 genes that are shared by all lobes and enriched in inflammatory pathways such as TNF-α signaling via NF-κb. The genome-wide H3K27ac data revealed lobe-specific patterns in the lobe behind the heart (H) and the left lobe (L) in the control and hPMSC groups, whereas these patterns were confused in the GVHD and PBS groups. Gene set enrichment analysis revealed that the hPMSC-induced variations in genome-wide H3K27ac were concentrated in the L and R3 lobes. The genes showing accordant tendencies (a-DEGs) between the transcriptome and H3K27ac highly overlapped between the a-DEGs and the Rein02 genes when hPMSCs were compared with GVHD. Integrated multiomics analysis suggested that the a-DEGs were predominantly expressed on myeloid (Fam174a, Ifi204, Slc7a11, Chil3, Capza2, Clec5a, and Clec4a2), T and NK cells (Eif3f, Cited2, Crybg1, Ndufs4, and Emb), B cells (Fam174a, Eif3f, and Blnk), and epithelial cells (Alcam, Chmp2b, and Metap2). The subset with high expression levels of these genes tended to present anti-inflammatory effects and reduced cytotoxic activity. Our study may provide new insights into the development of potential therapeutic drugs that target H3K27ac to assist in MSC treatment.

## 1 Introduction

Hematopoietic cell transplantation (HCT) provides curative therapy for many hematological malignancies, but its clinical applications are limited primarily by graft-*versus*-host disease (GVHD) (1). Current studies have focused on the ability of GVHD to target the intestines, liver, and skin, probably because damage (diarrhea, increased bilirubin, rash, *etc.*) to these organs is relatively easy for clinicians to detect (2). However, few studies have focused on the ability of GVHD to target other nonclassic organs, such as the central nervous system, lungs, ovaries and testis, thymus, bone marrow, and kidney. A better understanding of GVHD and treatments for nonclassical GVHD target organs may help improve patient outcomes after allo-HSCT. While glucocorticoids remain the only standard initial treatment for acute GVHD (3), mesenchymal stem cells (MSCs) have been shown to be effective treatments for GVHD because of their immunosuppressive activity. MSCs are present in all tissues, play key roles in maintaining tissue homeostasis and usually possess significant immunosuppressive activities (4). The immunomodulatory properties of allogeneic MSCs allow them to be employed to limit and treat acute GVHD (aGVHD) after HCT. To date, much remains unknown about the molecular characteristics and pathological conditions of aGVHD in the lung after MSC treatment.

Epigenomic regulation through histone modification has been implicated in numerous diseases, such as tumors and autoimmune diseases. Acetylation of lysine 27 of histone H3 (H3K27ac) is well recognized as a marker for active enhancers (5, 6). Numerous studies have indicated the important role of H3K27ac in the inflammatory response (7–13). However, few studies have investigated H3K27ac variations in response to MSCs in the lungs of patients with aGVHD. Moreover, little is known about whether there are differences among different lung lobes regarding this issue. In the present study, human placenta-derived MSCs (hPMSCs) were proposed to affect the genome-wide H3K27ac landscape among different lung lobes suffering from aGVHD, which subsequently triggered different key genes involved in anti-inflammatory pathways in different lung lobes. Here, cleavage under targets and tagmentation (CUT&Tag), which has more advantages than ChIP-seq, was used to compare the profiling of H3K27ace in normal mice, GVHD mice, GVHD mice treated with PBS, and GVHD mice treated with hPMSCs. Inflammation-related signaling pathways were synchronously profiled through bulk RNA-seq. Our study provides the first understanding of the epigenomic regulatory effects of MSCs on aGVHD in the lungs, which may provide insight into the pathogenesis of nonclassic GVHD target organs and lead to the development of prevention and treatment measures.

## 2 Materials and methods

### 2.1 Ethical statement

Animal experimental protocols designed in strict accordance with the guidelines, as well as the preparation of hPMSCs, were reviewed and approved by the research ethics committee of Binzhou Medical University (BZMU2021–163).

### 2.2 Preparation of hPMSCs, murine models and tissue collection

**Figure 1:**
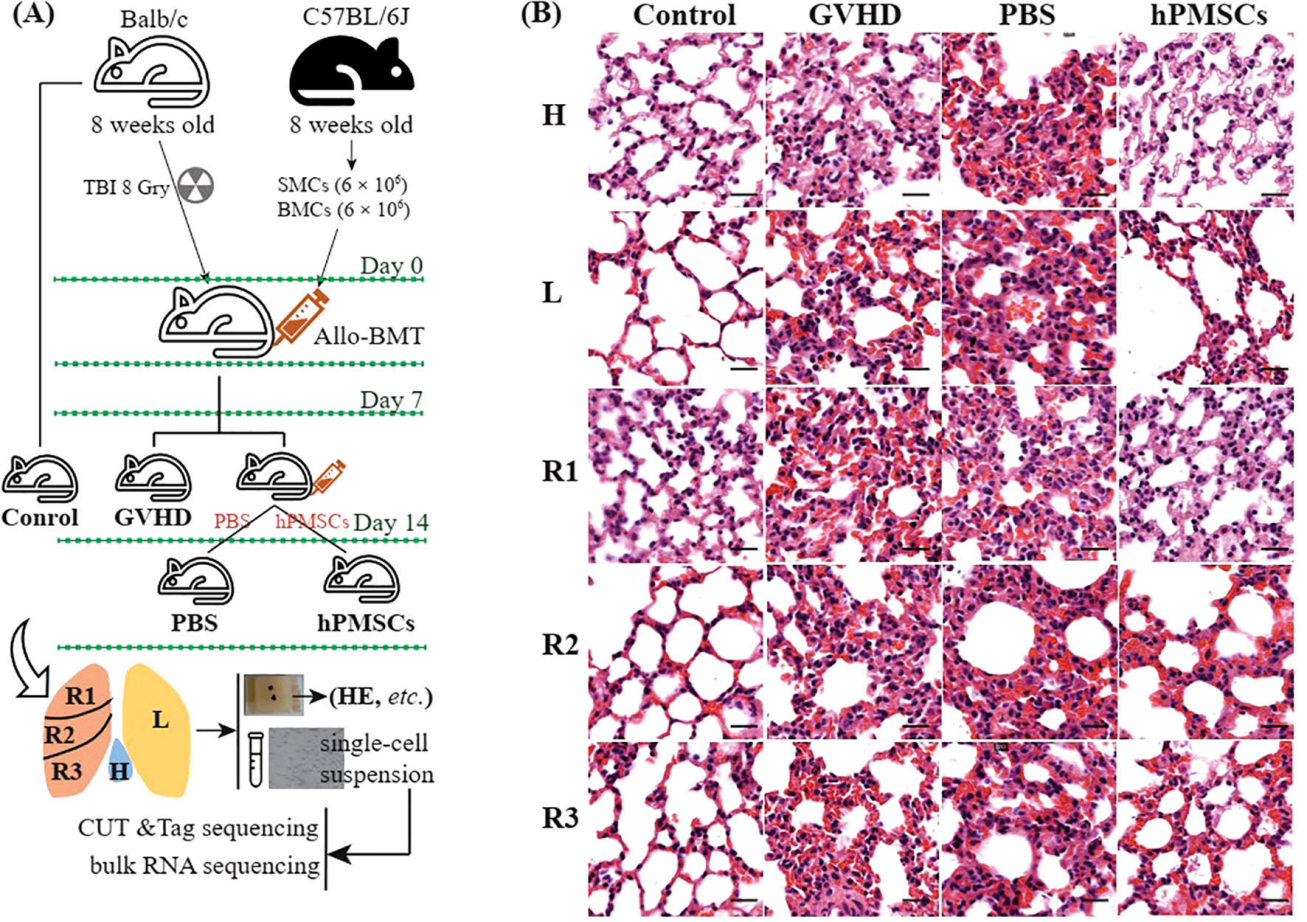
Schematic diagram of the experimental design **(A)** and HE staining of the lung lobes **(B)**. Scale bar, 20 μm. H, lobe behind the heart; L, left lobe; R1, upper right lobe; R2, middle right lobe; R3, lower right lobe.

hPMSCs were isolated as described in our previous study (14). A total of 12 female BALB/cj mice in the same batch were included in this study. As shown in **Figure 1A**, three mice without any treatment were assigned to the control group, and 9 of the 12 mice were used to construct GVHD model mice as described in our previous study (14): they were irradiated with 8 Gy whole-body X-ray irradiation and then transplanted with donor bone marrow cells (6 ×10^6^) and splenic mononuclear cells (6× 10^6^) from male C57BL/6j *via* the tail vein the next day. On day 7 after transplantation, 100 μL of PBS (PBS group, n = 3) or 100 μL of PBS mixed with hPMSCs (hPMSC group, n = 3) was injected into GVHD mice through the tail vein for intervention treatment. The remaining three GVHD mice (GVHD group, n = 3) and the control group were sacrificed for immediate harvesting of the lung lobes via cervical dislocation after anesthetization. On day 14 after transplantation, the mice in the PBS and hPMSC groups were sacrificed for immediate harvesting of the lung lobes. For each lung lobe, one half was processed into a single-cell suspension via a standardized protocol as previously described (15), followed by CUT&Tag and bulk RNA-seq analysis; the other half was fixed with 4% formalin, embedded in paraffin, sliced, and stained with hematoxylin and eosin (HE). The slides were scanned on a Pannoramic SCAN (3DHISTECH. Ltd, Hungary). Weight loss, posture, activity, fur texture, skin integrity, and diarrhea were scored on a severity scale of 0–2 as described previously (16).

### 2.3 Constructions of CUT&Tag and mRNA-seq libraries

The CUT&Tag and mRNA-seq libraries were constructed as described previously (17). In brief, a total of 1 ×10^5^ cells and 1×10^6^ cells in each sample were processed to construct the CUT&Tag library via the Hyperactive^®^ Universal CUT&Tag Assay Kit (Vazyme Biotech Co., Ltd., China) and the mRNA-seq library via the VAHTS^®^ Universal V8 RNA-seq Library Prep Kit for Illumina (Vazyme Biotech Co., Ltd.), followed by sequencing on a NovaSeq 6000 platform in Novogene (Beijing, China). The sequencing datasets have been deposited in the National Genomics Data Center (https://ngdc.cncb.ac.cn/) under the login number CRA011355.

### 2.4 Data analysis

#### 2.4.1 Read processing

As described in our previous study (17), the raw reads in the CUT&Tag libraries were filtered via fastp software and aligned against the GRCm39 genome dataset and the *E. coli* genome with Bowtie2, followed by spike-in calibration, removal of duplicates with Picard tools, coverage normalization and assessment of the fragment size distribution via SAMtools, calling of peaks via the SEACR tool, and peak annotation via the ChIPseeker package. For the reads in the mRNA-seq libraries, the raw reads were filtered via fastp software, and subsequently, the subjunc aligner and featureCounts in Subread software (18) were used to align the clean reads against the GRCm39 genome dataset and construct the gene–sample count table.

#### 2.4.2 Variation trends in gene expression

For each lobe, the differential gene sets (DEGs) $G_{i}^{j}$ were calculated via pairwise comparisons among the GVHD group, control group, PBS group and hPMSC group via the function “DESeq” in the DESeq2 package (19). that is,


$$G_{i}^{j}=DESeq\left(j/i\right)$$

where *i*, *j ∈ (GVHD group, control group, PBS group, hPMSC group)*, *DESeq(j/i)* calculates the significant fold change in the differential gene ( $g_{i}^{j}$ ) for numerator *j* vs denominator *i*. For $g_{i}^{j}$ in $G_{i}^{j}$ ,


$$\{\begin{array}{c} g_{i}^{j}=\;g_{i}^{j},\;if\;p.adj\leq 0.05\\ g_{i}^{j}=0,\;if\;p.adj>0.05\; \end{array}$$

Then, $g_{i}^{j}$ in different $G_{i}^{j}$ s was used for variation trends of gene expression.

#### 2.4.3 Functional enrichment analysis

In this study, we designed four schemes to mine the data. The schemes are shown in the corresponding figures. The clusterProfiler package was used to perform gene set enrichment analysis (GSEA) and gene set variation analysis (GSVA) against the hallmark gene set in MSigDB and the T-cell function gene set as described in our previous study (20–22). The intersections among different comparisons were performed via the UpSetR package. The STRING database was used to predict protein–protein interactions.

#### 2.4.4 Single-cell RNA dataset processing

The single-cell datasets GSM6758168, GSM6758169, GSM6758170, GSM6758179, GSM6758180, GSM6758181, and GSM6711820 were downloaded from the GEO database and then processed as follows. Although ALI/ARDS and GVHD have distinct etiologies, both conditions share hyperinflammatory states characterized by elevated TNF-α, IL-6, and GM-CSF levels, as well as pathogenic Th17 expansion (12–18% in GVHD *vs.* 10–15% in ALI/ARDS), providing a relevant model for studying inflammatory responses in the lung (23, 24). The cells were flagged as poor quality if they met one of the following thresholds: 1) number of features (nFeature_RNA) < 400 or nFeature_RNA > 7,000; 2) number of reads (nCount_RNA) < 500 or nCount_RNA > 80,000; and 3) total percentages of mitochondrial genes > 15% or ribosomal genes > 40%. The mitochondrial and ribosome genes were removed, the cell cycle phases were scored, and SCT transformed the expression of all the genes on the basis of the scores of the cell cycle phases. After executing the “RunPCA”, “FindNeighbors”, “FindClusters”, “RunTSNE” and “RunUMAP” functions, cluster annotation was manually performed on the basis of the cell markers. The cell markers used were Epcam, Krt19, Krt18 and Cdh1 for epithelial cells; Dcn, Thy1, Col1a1 and Col1a2 for fibroblasts; Pecam1, Cldn5, Flt1 and Ramp2 for endothelial cells; Cd3d, Cd3e, Cd3g and Trac for T cells; Nkg7, Gnly, Ncam1 and Klrd1 for NK cells; Cd79a, Ighm, Ighg3 and Igha2 for the B-cell lineage; Lyz, Marco, Cd68, and Fcgr3a for myeloid cells; and Kit and Ms4a2 for mast cells. The function “FindMarkers” with the parameter “min.pct = 0.3” was employed to detect the differentially expressed genes (DEGs).

#### 2.4.5 Statistical analysis and plots

For the bioinformatic data, the built-in tests of the software were used by default. The software automatically calculates adjusted p values, with p < 0.05 considered statistically significant. The ggplot2 package was employed to plot bar plots and scatter plots. The ComplexHeatmap package was employed to plot heatmaps.

## 3 Results

### 3.1 Transcriptomic profiles in different lung lobes

**Figure 2:**
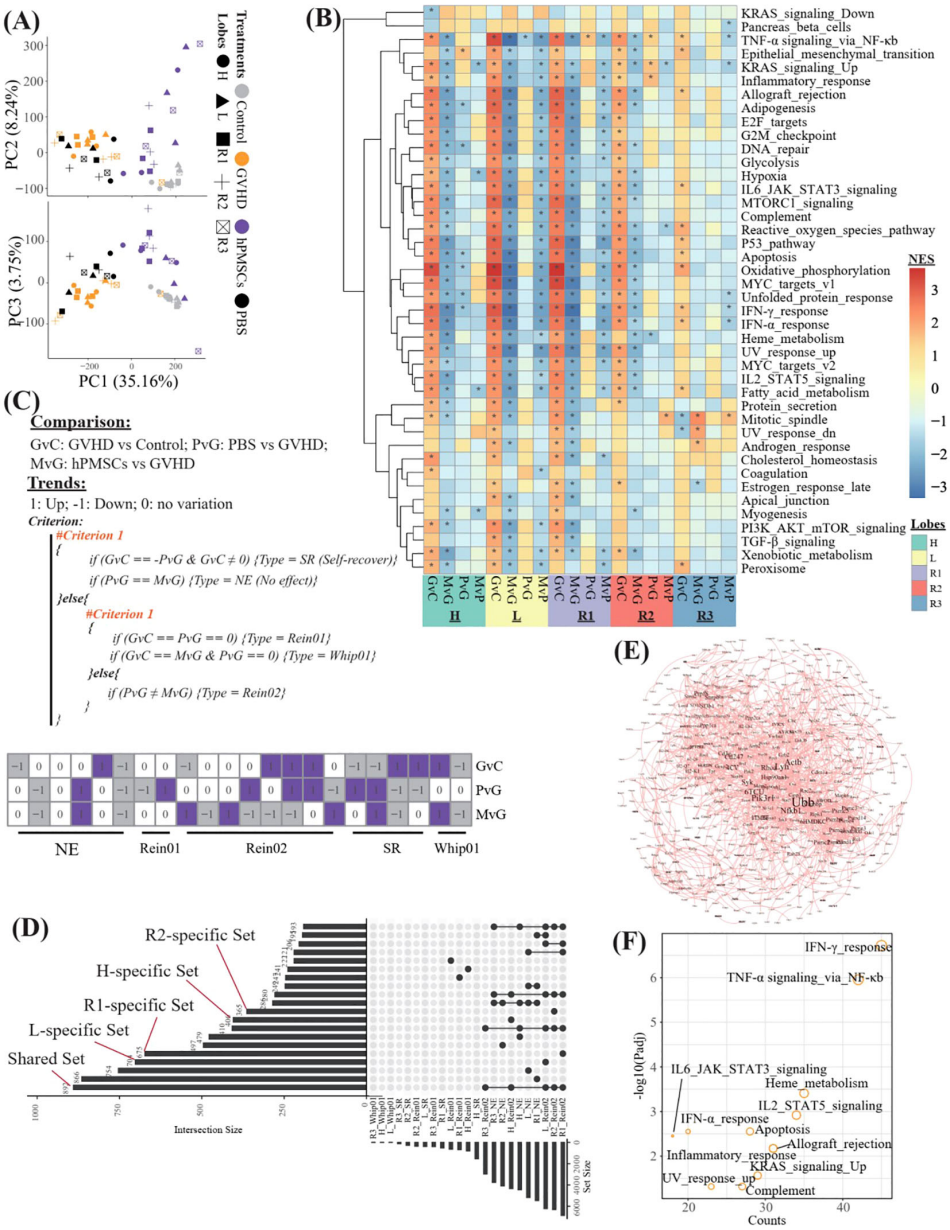
Transcriptomic profiles of different lung lobes. **(A)** Principal component analysis (PCA) of all samples on the basis of the transcriptome. **(B)** Heatmap showing the normalized enrichment scores (NESs) of GSEA against the hallmark gene set of MsigDB on the basis of the DEGs derived from different comparisons in different lobes. G, M, P, and C represent the GVHD, hPMSC, PBS, and control groups, respectively, and the letter “v” concatenating them means “vs”. GvC refers to a comparison between GVHD patients and controls in a similar fashion. **(C)** Algorithm used to identify the types of DEGs across different comparisons, and finally, a total of 5 types of DEGs were assigned. **(D)** UpSet plot illustrating the intersections across different types of DEGs in different lobes. “R1_Rein02” refers to the DEGs assigned to the Rein02 type in the R1 lobe. “L_specific Set” refers to the Rein02-type DEGs that occurred only in the L lobe. “Shared set” refers to the Rein02-type DEGs that occurred in all lobes, and so on, in a similar fashion. **(E)** Protein–protein interaction network of the DEGs in the shared set. **(F)** Enrichment of the DEGs in the shared set against the hallmark gene set of MsigDB. The symbol * indicates statistically significant P-values in the Normalized Enrichment Score (NES).

The study schemes are illustrated in **Figure 1A**. Compared with those in the GVHD and PBS groups, HE staining revealed that hPMSC treatment reduced inflammatory infiltration, thinned the alveolar wall, and improved bronchiolar epithelial edema in all lobes. At the transcriptomic level, the first 3 axes of the PCA presented a total of 47.15% of the variance (**Figure 2A**). In the PC1 axis, the samples were clustered into two groups: one group was composed of samples from the control and hPMSC groups, and the other group was composed of samples from the GVHD and PBS groups, demonstrating recovery trends of hPMSCs toward the control group. The hPMSC samples were likely grouped into the L subgroup or the R1 and R2 subgroups, whereas the samples in the other groups were undistinguishable among the lobes (**Supplementary Figure S1**).

On the basis of the DEGs derived from different comparisons (GvC, MvG, PvG and MvP) in different lobes, we performed GSEA against the hallmark gene set of MsigDB and identified 42 varied pathways (**Figure 2B**). The R3 lobe presented the lowest number of varied pathways in all lobes, and in the R3 lobe, the comparison of GVHD vs control (GvC) revealed the greatest number of varied pathways within the R3 comparisons. In general, in all lobes, the pathways upregulated in GvC were always downregulated in MvG, and only a few of them, especially the pathway “TNF-α signaling via_NF-κb”, remained significantly upregulated in PvG. To further identify the potentially key genes involved in the ability of MSCs to treat GVHD, we propose the algorithm described in **Figure 2C**, which aims to assign all the DEGs into 5 types, including genes with no effects (NE), genes likely involved in the therapeutic effects of MSCs (Rein01 and Rein02), genes involved in self-recover (SR), and genes potentially exacerbating GVHD (Whip01). We then focused on the DEGs of the Rein02 type because they presented trends in MvG in contrast to those in PvG or GvC. As shown in **Figure 2D** and **Supplementary Table 1**, 892 Rein02 genes were shared by all the lobes, whereas 406, 704, 675, 365, and 41 genes were specifically expressed in the H, L, R1, R2 and R3 lobes, respectively. The protein–protein interaction network of the 892 shared genes is shown in **Figure 2E**, highlighting the high centrality degrees of Nfkb1, Ubb, and Lyn. Further enrichment of the 892 shared genes against the hallmark gene set revealed that the top 5 enriched pathways were the IFN-γ response, TNF-α signaling via NF-κb, heme metabolism, IL2/STAT5 signaling, and allograft rejection, revealing the predominant target pathways of MSCs in treating GVHD across all lobes.

### 3.2 Genome-wide profiles of H3K27ac in all lobes

**Figure 3:**
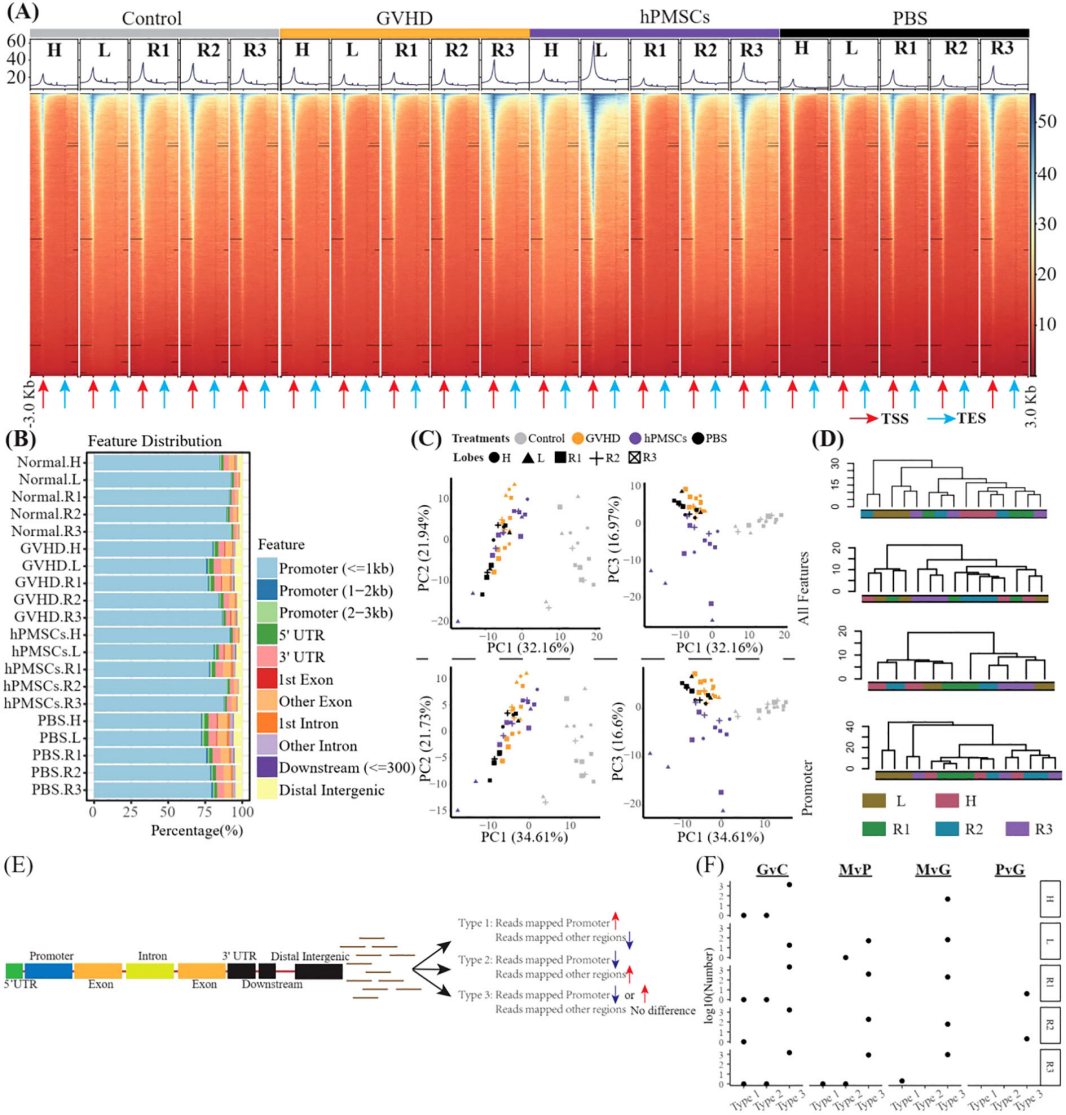
CUT&Tag captures genome-wide profiles of H3K27ac in all lobes. **(A)** Heatmaps showing the genomic occupancy of H3K27ac ± 3 kb flanking TSSs and TESs. **(B)** Genome-wide distribution of H3K27ac binding regions. **(C)** PCA was performed on all groups of total H3K27ac and promoter H3K27ac. **(D)** Dendrograms presenting the hierarchical cluster of lobes on the basis of all the features of H3K27ac in the genome in each group. **(E)** Algorithm scheme to identify genes with a shift in H3K27ac from promoter regions to other regions. **(F)** Scatter plot illustrating the number of genes mentioned in E in all comparisons of all lobes. G, M, P, and C represent GVHD, hPMSC, PBS, and the control group, respectively, and the letter “v” concatenating them means “vs”. GvC is a comparison between the GVHD and control groups, and so on, in a similar fashion.

We then profiled the genome-wide alterations in H3K27ac in all groups of all lobes via CUT&Tag sequencing. The peak fragment size distribution of approximately 200 bp indicated successful CUT&Tag experiments (**Supplementary Figure S2**). The concentration maps revealed that the peak-out enrichment regions of H3K27ac were concentrated in the TSS region, and similar peak distributions were observed between the hPMSC and control groups and between the GVHD and PBS groups (**Figure 3A**). Further gene annotation identified 5,896 relevant genes and revealed that most of the H3K27ac-modified reads were concentrated in the promoter region (**Figure 3B**). However, the percentages of promoter binding sites were significantly reduced in the GVHD and PBS groups, especially in the H, L and R1 lobes, suggesting that disordered H3K27ac modifications in the genome in these lobes were likely caused by GVHD. Compared with the control, hPMSCs exhibited recovery features. PCA on the basis of both the total features and the promoter features revealed that the distance from hPMSCs to the control group was shorter than that from the GVHD and PBS groups to the control group on the PC3 axis (**Figure 3C**). PCA based on promoter features presented the same pattern as PCA based on total features, indicating that variations in promoter regions predominated the genome-wide alteration of H3K27ac modifications. In the control group, hierarchical clustering based on all the features of H3K27ac broadly grouped the lobes into H, L, and right lobes (R1, R2, R3), which were confused by GVHD and PBS (**Figure 3D**). hPMSCs restored the L lobe.

We then wondered whether there was a gene with a shift in H3K27ac from the promoter region to other regions. That is, for one gene, the number of reads mapped to the promoter region increased or decreased, whereas the number of reads mapped to other regions decreased or increased, respectively (**Figure 3E**). As shown in **Figure 3F**, only 8 genes were Type 1 (Mrpl3, Cdk14 and Lpl) and Type 2 (Vrk2, Rad51b, Cdyl, 4930543E12Rik and Gm35307), whereas Type 3 genes predominated. Moreover, within different lobes, the number of Type 3 genes differed.

**Figure 4:**
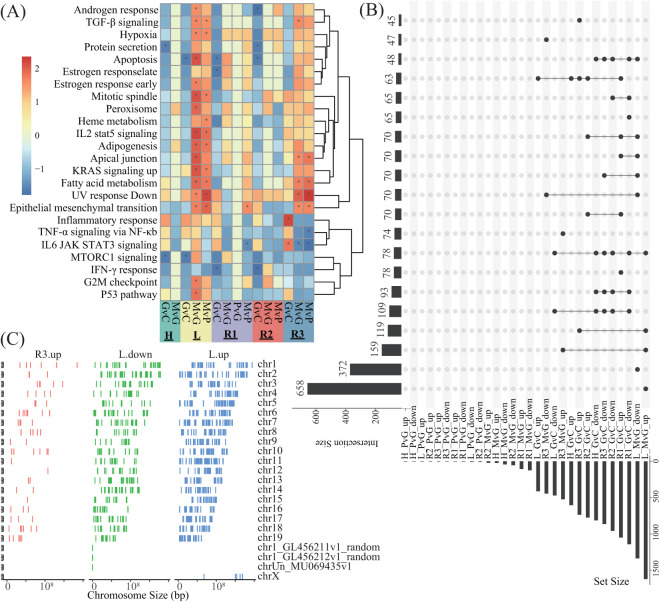
The enrichment analysis of the DEGs and their intersections and their distributions in the genome. **(A)** Heatmap showing the NES of GSEA against the hallmark gene set of MsigDB on the basis of the promoter DEGs of different comparisons in different lobes. G, M, P, and C represent the GVHD, hPMSC, PBS, and control groups, respectively, and the letter “v” concatenating them means “vs”. GvC indicates a comparison between GVHD patients and controls in a similar fashion. **(B)** UpSets plot illustrating the intersections across different types of promoter DEGs of different comparisons in different lobes; up- and downlayers indicate upregulated and downregulated DEGs, respectively. **(C)** Genome-wide distribution of the DEGs specifically observed in L_MvG_up (L.up), L_MvG_down (L.down) and R3_MvG_up (R3.up).

The promoter DEGs identified in all the comparisons across all the lobes are shown in **Supplementary Figure S3**. GSEA against the hallmark gene set of the MsigDB revealed that the significantly enriched pathways were predominantly associated with MvG and MvP in the L and R3 lobes, whereas no significantly enriched pathways were associated with PvG in any lobe (**Figure 4A**). Similarly, as shown in **Figure 4A**, the majority of DEGs were found in the MvG of the L and R3 lobes and in GvC in all lobes, whereas the number of DEGs in PvG was low. The MvG- or GvC-derived upregulated or downregulated genes presented a high number of accordant intersections between the L and R3 lobes, whereas the other intersections presented relatively lower numbers of DEGs. Notably, a total of 658 and 372 upregulated DEGs and downregulated DEGs were specifically observed in the MvG of the L lobe, and 74 upregulated DEGs were specifically observed in the MvG of the R3 lobe. In addition, these genes did not present chromosomal bias (**Figure 4C**).

### 3.3 Integrated analysis of bulk RNA-seq, CUT&Tag and scRNA-seq data

**Figure 5:**
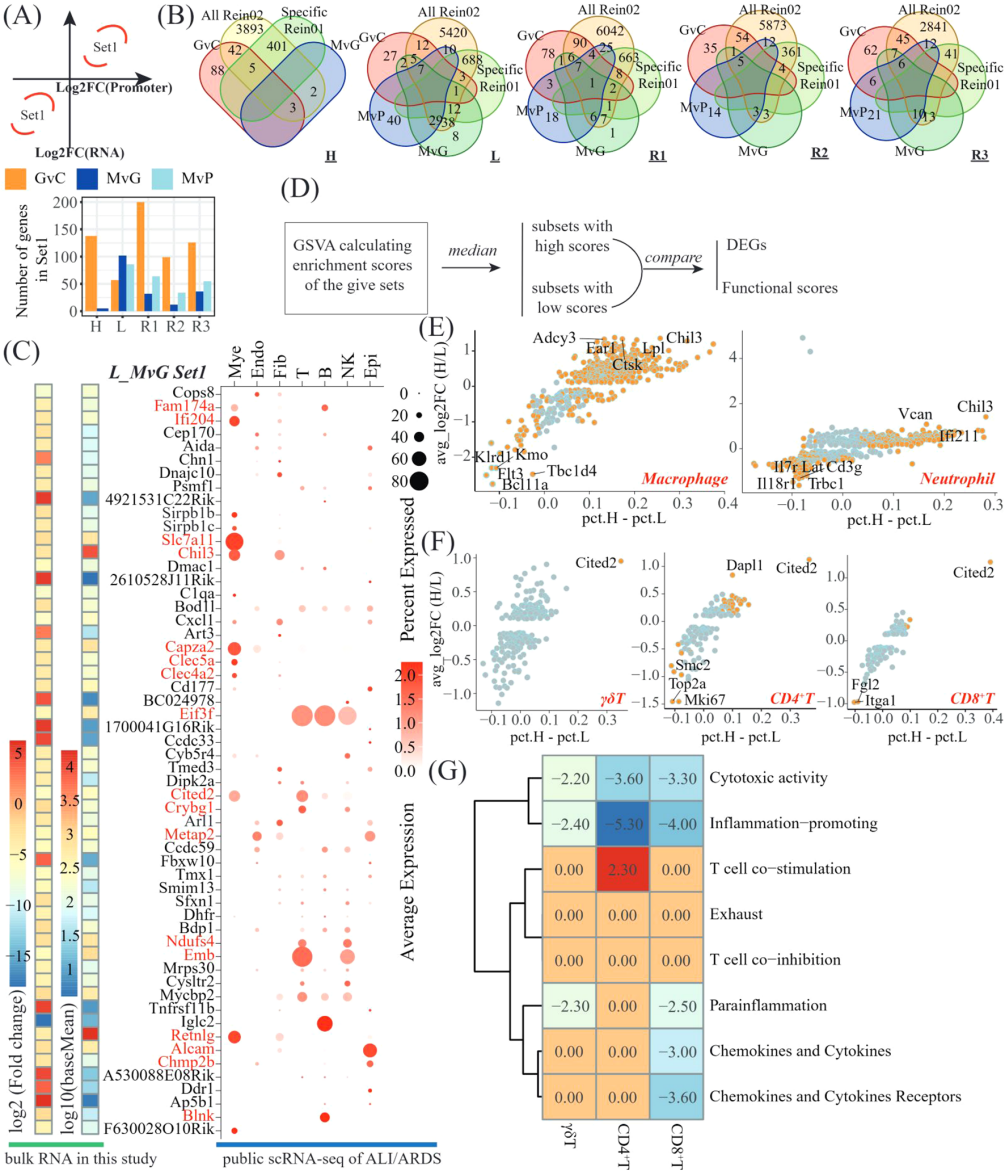
Integrated analysis revealing the suppressive genes involved in hPMSC treatment of L-lobes. **(A)** Scheme to identify the genes showing accordant tendencies in bulk RNA-seq and CUT&Tag seq (top) and bar plots showing the number of accordant genes (a-DEGs) in different comparisons of different lobes (bottom); **(B)** Venn diagram showing the intersections among the a-DEGs of the GvC, MvG and MvP genes, the shared Rein02 genes, and the lobe-specific Rein02 genes; **(C)** Heatmap showing the average expression and log2-fold change of a-DEGs of MvG in the L lobe and the dot plots presenting the expressing cells of these a-DEGs in GSM6711820; **(D)** Scheme of investigations on whether the a-DEGs of MvG in the L lobe were suppressive genes; Scatter plots illustrating the DEGs mentioned in 5D in macrophages and neutrophils **(E)** and γδT, CD4^+^T cells and CD8^+^T cells **(F)**; **(G)** Heatmap displaying t statistics derived from pairwise comparisons of GSVA scores for T-cell functional pathways within each T-cell subset (γδT, CD4^+^T, and CD8^+^T cells). For each subset, the cells were stratified into high- and low-expression subgroups on the basis of signature gene expression. GSVA scores were compared between subgroups via t tests, with t values representing the magnitude and direction of pathway activity differences. Adjusted p values > 0.05 were filtered by setting the corresponding t values to zero.

We then focused on the genes showing accordant tendencies in bulk RNA-seq and CUT&Tag seq (**Figure 5A**). As observed above, only promoter-related comparisons in CUT&Tag-seq were included. The accordant genes (a-DEGs) are listed in **Supplementary Table S2** and summarized in **Figure 5B**. No a-DEG was found in the PvG in any lobe. The L lobe presented the greatest number of a-DEGs in MvG but the lowest number of a-DEGs in GvC. The intersections of the a-DEGs with the Rein02 genes were revealed (**Figure 5C**). Notably, most of the a-DEGs in MvG were Rein02 genes in all lobes except for the H lobe. We subsequently explored the cells expressing the a-DEGs in the MvG of the lobe by resuring publicly available single-cell RNA sequencing datasets of murine lungs with acute lung injury and acute respiratory distress syndrome (ALI/ARDS), which were induced via a two-hit approach combining lipopolysaccharide-induced inflammation and mechanical ventilation-induced injury. After filtering by average expression, 56 out of 102 a-DEGs were investigated (**Figure 5D**). We identified sixteen highly expressed DEGs via both the bulk RNA-seq data of this study and the public scRNA-seq data of ALI/ARDS patients, and these genes were significantly upregulated in the MvGs of the L lobe. These a-DEGs were predominantly expressed on myeloid (Fam174a, Ifi204, Slc7a11, Chil3, Capza2, Clec5a, and Clec4a2), T and NK cells (Eif3f, Cited2, Crybg1, Ndufs4, and Emb), B cells (Fam174a, Eif3f, and Blnk), and epithelial cells (Alcam, Chmp2b, and Metap2). Here, these genes were provisionally termed signature gene sets in the downstream analysis. Similar findings have been reported in other public datasets (**Supplementary Figure S4**).

Given the anti-inflammatory effects of MSCs and the upregulation of these genes, we wondered whether these signature genes are suppressive genes in the lung immune system. We designed a scheme based on the merged dataset of seven public scRNA-seq datasets of ALI/ARDS patients (**Figure 5D**, **Supplementary Figure S5**). Compared with the subsets with low expression of signature genes, the top 3 upregulated genes in the subsets expressing high-level signature genes were Ear1, Chil3, and Ctsk in macrophages and Chil3, Can and Ifi211 in neutrophils (**Figure 5E**). In T cells, the CD4^+^ T cells presented the greatest number of DEGs, with the top 3 upregulated genes being Cited2, Dapl1, and Itgb3 and the top 3 downregulated genes being Mki67, Top2a and Stmn1 (**Figure 5F**). Moreover, by employing GSVA against T-cell functional gene sets, we observed that cytotoxic activity and inflammation promotion were significantly impaired in the subsets of γδT, CD4^+^T and CD8^+^T cells expressing high levels of signature genes (**Figure 5G**). In addition, parainflammation, chemokine and cytokine expression, and chemokine and cytokine receptor expression were impaired in the subsets of CD8^+^ T cells expressing high levels of signature genes.

## 4 Discussion

In this study, we described variations in the transcriptome and genome-wide H3K27ac modifications in the treatment of GVHD with hPMSCs in different lung lobes. Unsurprisingly, HE staining demonstrated the therapeutic effect of hPMSCs on GVHD in all lung lobes (**Figure 1B**). In addition, the transcriptomic variations in all the lung lobes revealed upregulated inflammatory pathways in all the lobes, for example, TNF-α signaling via NF-κb, IL6 JAK STAT3 signaling, the IFN-γ response, the reactive oxygen species pathway, and allograft rejection, which play proinflammatory roles in recipients with GVHD (14, 25–27). hPMSC treatment downregulated these pathways in all lobes. Moreover, the algorithm shown in **Figure 2C** identified a set of genes whose expression was downregulated by hPMSCs to prevent degeneration and whose expression was shared by all lobes. The enriched pathways of these genes were also the above inflammatory pathways (**Figure 2F**), suggesting that the pathways underlying GVHD progression in the lung are the same as the pathways in classical organs. In **Figure 2E**, the genes Nfkb1, Ubb, and Lyn are highlighted because of their central roles in the protein–protein interaction network. These findings align with the central roles of NF-κB, ubiquitination processes mediated by Ubb, and Lyn kinase in regulating inflammatory cascades. NF-κB is a master regulator of inflammation and immune responses and is directly linked to the enriched TNF-α signaling via the NF-κB pathway observed in our study. Its centrality implies that hPMSC therapy may suppress GVHD-driven hyperinflammation by modulating NF-κB activity, thereby reducing TNF-α/IL-1β production and T-cell activation (28, 29). Ubiquitination regulates protein degradation, immune signaling, and stress responses. The high centrality of Ubb suggests that hPMSC treatment may stabilize or degrade key immune regulators to control inflammatory cascades in GVHD (30). Lyn regulates B-cell receptor signaling and myeloid cell functions through interactions with the IL2/STAT5 and cytokine signaling pathways (31). These findings suggest that hPMSCs may modulate Lyn-dependent pathways to inhibit donor T-cell activation or macrophage polarization, which aligns with the observed suppression of allograft rejection pathways.

However, little is known about the role of genome-wide H3K27ac in GVHD progression and MSC treatment, even in classical organs. Since drugs targeting H3K27ac have been applied in the clinical treatment of cancers (32, 33), uncovering the variations in H3K27ac modifications in GVHD and MSC treatment might provide new insights and potential therapeutic drugs for treating GVHD. In this study, we demonstrated significant variations in genome-wide H3K27ac modifications in the GVHD group compared with the control group and in the hPMSC group compared with the GVHD group but not in the PBS group compared with the GVHD group. These observations demonstrated that GVHD significantly altered the genome-wide H3K27ac modification of lung lobes. The greater similarity between the hPMSC and control groups than between the GVHD, PBS and control groups suggested that hPMSCs may exert therapeutic effects through dynamic H3K27ac reconfiguration to partially restore homeostatic chromatin states. This epigenetic restoration likely contributes to the alleviation of GVHD. In this study, we observed a reduction in H3K27ac modifications in promoter regions in the GVHD groups. In addition to the promoter regions, H3K27ac could be enriched in the 1^st^ intron region to act as a marker of a superenhancer (SE) (34). We then wondered whether there was a shift in H3K27ac from the promoter to other regions in one gene, which might explain the reduction in H3K27ac in the promoter. However, very little shift within the same gene was detected. In addition, no significant hPMSC-induced chromosomal bias was detected. While our study focused on H3K27ac, histone modifications often act in concert to regulate chromatin states (35). The depletion of H3K27ac at promoters may coincide with gains in repressive marks such as H3K27me3 or losses in activating marks such as H3K4me3, synergistically repressing transcription (36, 37). For example, the Chil3 promoter exhibited reduced H3K27ac in GVHD, which could be correlated with increased H3K27me3 to silence this anti-inflammatory gene. However, our study did not profile other histone marks, limiting mechanistic insights. Notably, the concordance between H3K27ac changes and transcriptomic shifts supports a direct regulatory role, potentially mediated by enhancer–promoter looping (38).

Surprisingly, the DEGs derived from the variations in H3K27ac in promoter regions, as well as the GSEA enrichment of these DEGs, indicated that the predominant hPMSC-induced variations in H3K27ac were concentrated in the L and R3 lobes (**Figures 4A, B**), which was in accordance with the observations that hPMSCs recovered H3K27ac modifications in the L and R3 lobes (**Figure 3D**). The lobe-specific H3K27ac alterations induced by hPMSCs may stem from anatomical and functional heterogeneity across the lung lobes. The L and R3 lobes exhibit distinct mechanical stresses due to their positioning and airflow dynamics, potentially influencing immune cell infiltration and epigenetic regulation during GVHD. Additionally, transcriptomic data revealed that inflammatory pathways were most dynamically regulated in the L and R3 lobes, suggesting that hPMSCs preferentially target regions with heightened inflammatory activity. Single-cell analysis further highlighted myeloid and T cells as key expressers of hPMSC-regulated genes, and the L/R3 lobes may harbor higher proportions of these cell populations, rendering them more responsive to epigenetic reprogramming (39, 40). This microenvironmental and cellular heterogeneity could collectively explain the lobe-specific effects of hPMSCs. The relatively low overlap between genes showing concordant trends in RNA-seq and H3K27ac modifications and the total number of DEGs can be attributed to the multilayered complexity of transcriptional regulation. While H3K27ac marks active enhancers, transcriptional output is influenced by the interplay of multiple histone modifications, such as H3K4me1 and H3K27me3, which can either synergize with or counteract H3K27ac effects (41). Additionally, chromatin modifications may precede transcriptional changes, and transient transcriptional bursts may not sustain detectable chromatin alterations (42, 43). Furthermore, enhancer–promoter interactions can regulate multiple genes, where a single enhancer marked by H3K27ac might activate one gene while repressing another, complicating direct correlations between H3K27ac and gene expression (44, 45).

To further explore the genes driven by H3K27ac, we focused on the genes showing accordant tendencies in bulk RNA-seq and CUT&Tag-seq (a-DEGs). The number of a-DEGs in MvG was much greater in the L lobe and R3 lobes than in the other lobes, which was in agreement with the assumption that hPMSCs recovered H3K27ac modifications mainly in the L and R3 lobes. The intersections of the a-DEGs and Rein02 genes revealed that the a-DEGs of MvG were predominantly Rein02 genes and were upregulated by hPMSCs (**Figures 5B, C**). Further analysis of the public scRNA-seq dataset revealed that these genes were predominantly expressed on myeloid and T cells. Previous studies have acknowledged the crucial roles of T cells in the use of MSCs to treat GVHD, especially the associated inflammatory pathways, including TNF-α signaling, IL6 signaling, the IFN-γ response, and ROS in donor T cells (46–48). In this study, the gene set of a-DEGs, including Eif3f, Cited2, Crybg1, Ndufs4, and Emb, was identified to be relatively specific for T cells. The CD4^+^ T-cell subset with high enrichment scores for this gene set presented higher levels of Dapl1 than did the subset with low enrichment scores. A recent study reported that Dapl1 could facilitate the dysfunction of exhausted CD8^+^ T cells in chronic infection and cancer (49), suggesting that hPMSCs might control GVHD through facilitating exhausted T cells *via* upregulated Dapl1. Moreover, the enrichment scores of the T-cell functional gene set demonstrated reduced cytotoxic activity and inflammation-promoting ability in the subsets with high enrichment scores of the signature gene set (**Figure 5G**). In myeloid cells, the a-DEG Ifi204 could negatively regulate the IRF7-mediated type I interferon response to avoid unnecessary host damage from hyperinflammatory responses (50). Chil3 was reported to promote M2 macrophage differentiation and has been shown to act as an anti-inflammatory agent (51). Capza2 has been implicated in reducing the inflammatory response of macrophages (52, 53). Moreover, the DEGs derived from the comparison of the subset with high enrichment scores to that with low enrichment scores for these genes presented higher levels of Ear1 and Ctsk, which have been identified to be closely associated with anti-inflammatory macrophages (54, 55). These observations suggest several potential mechanisms by which hPMSCs alleviate GVHD in the lungs, particularly in the L lobe.

Numerous studies have reported diverse and complex mechanisms by which MSCs treat GVHD, with causal associations with multiple types of cells and molecules. This study also suggested potential mechanisms associated with several types of cells and molecular pathways. However, the multiple possible mechanisms suggested in this study make further verification via laboratory and clinical experiments difficult. Therefore, our study is currently a prospective study and inefficient in further examinations, and the possible mechanisms suggested in this study should be examined one by one in the future. Even so, our descriptions of genome-wide H3K27ac and the suggested cells and molecules implicated in GVHD and MSC treatment should provide useful information for further exploration of GVHD in the lungs and MSC treatment. Dapl1, which is implicated in T-cell exhaustion, could be targeted to increase hPMSC efficacy by stabilizing immunosuppressive T-cell subsets (49, 56). Similarly, Chil3, which promotes M2 macrophage polarization, might serve as a biomarker for monitoring hPMSC-driven immunomodulation. Pharmacological agents that modulate H3K27ac, such as HDAC inhibitors, could synergize with hPMSCs to reinforce anti-inflammatory effects (57, 58). Furthermore, lobe-specific H3K27ac signatures in circulating immune cells may serve as noninvasive biomarkers for GVHD progression. These strategies warrant validation in preclinical models to advance toward clinical trials.

In conclusion, our study described the paired profiles of the transcriptome and genome-wide H3K27ac in five lung lobes of the control group, GVHD group, hPMSC-treated group and PBS-treated group. We observed similar relevant mechanical pathways in all lobes as in other classical organs. However, the genome-wide H3K27ac in the control and hPMSC groups exhibited lobe-specific patterns. Notably, hPMSC-induced H3K27ac was concentrated in the L and R3 lobes. Integrated multiomics analysis revealed several target genes in T cells and macrophages, such as Dapl1 in T cells and Chil3, Capza2 and Ctsk in myeloid cells.

## Data Availability

The datasets presented in this study can be found in online repositories. The names of the repository/repositories and accession number(s) can be found in the article/**Supplementary Material**.
