# Supplementary material for: Multiomics profiles of genome-wide alterations in H3K27ac in different lung lobes after acute graft-versus-host disease with MSCs treatment
Source: Front Immunol. 2025 May 15;16:1570916. doi: 10.3389/fimmu.2025.1570916 (PMC12119469; doi:10.3389/fimmu.2025.1570916)
Supplement: Supplementary file 1 [file DataSheet1.zip › Figure4.Function/DiffTablst2Upset.docx]

DiffTablst2Upset<-function(diffLstFromExtractAbsDiff){

###functions

dd<-function(x){

V<-ifelse(x != 0,ifelse(x>0,"Up","Down"),"NE")

n<-which(V != "NE")

V<-V[n]

names(n)<-V

return(n)

}

dd2<-function(tab,g){

x<-as.numeric(tab[,g])

n<-dd(x)

upn<-n[which(names(n) == "Up")]

downn<-n[which(names(n) == "Down")]

upgenes<-rownames(tab)[upn]

downgenes<-rownames(tab)[downn]

return(list(upgenes=upgenes,downgenes=downgenes))

}

diffLst<-diffLstFromExtractAbsDiff

L1<-names(diffLst)

outRes<-vector("list")

for(l in L1){

dtab<-diffLst[[l]]$mergedTab_all_sig

g1Types<-dd2(dtab,1)

g2Types<-dd2(dtab,3)

g3Types<-dd2(dtab,2)

outRes[[paste(l,"GvC","up",sep="_")]]<-g1Types$upgenes

outRes[[paste(l,"GvC","down",sep="_")]]<-g1Types$downgenes

outRes[[paste(l,"PvG","up",sep="_")]]<-g2Types$upgenes

outRes[[paste(l,"PvG","down",sep="_")]]<-g2Types$downgenes

outRes[[paste(l,"MvG","up",sep="_")]]<-g3Types$upgenes

outRes[[paste(l,"MvG","down",sep="_")]]<-g3Types$downgenes

}

return(outRes)

}
