# Supplementary material for: Multiomics profiles of genome-wide alterations in H3K27ac in different lung lobes after acute graft-versus-host disease with MSCs treatment
Source: Front Immunol. 2025 May 15;16:1570916. doi: 10.3389/fimmu.2025.1570916 (PMC12119469; doi:10.3389/fimmu.2025.1570916)
Supplement: Supplementary file 1 [file DataSheet1.zip › Figure4.Function/ExtractAbsDiff_withoutpadj.docx]

ExtractAbsDiff_withoutpadj<-function(countData,

colDta,

SampleCol,

MajorGroupLevel,

CompareGroupLevel,

ImmuneFunctionSets=NA,

T0="normal",T1="gvhd",T2A="msc",T2B="pbs",

connector="_",

sig=0.05,

RefSet="M",

gseaplotType="pvalue",

GSEAmaxoverlap=100,

ntop=20){

###参数解释

## countData,colDta,同DESeq

## SampleCol 指定sample名字的列名，或者是第几列

## MajorGroupLevel,确认比较固定在哪些层级，CompareGroupLevel则为，在同一层级内，比较发生在哪些样本之间。

### 注意，二者都是列名，或者第几列

### 比如本例中要比较同一肺叶在不同处理组之间，则 MajorGroupLevel为肺叶，CompareGroupLevel则为处理组

### connector,两个level之间的连接符

###T0，正常组，发病前

###T1，发病组

###T2a，有效治疗方式A

###T2b，对照治疗方式B

###T0,T1,T2A,T2B，均应为batchDiff中涉及的分组名，例如本例中的gvhd,normal,msc,pbs

require(DESeq2)

require(dplyr)

ModiTab<-function(tab){

v1<-tab$log2FoldChange

v2<-tab$pvalue

v3<-tab$padj

v1<-ifelse(v3>0.05|is.na(v3),0,v1)

tab$log2FoldChange<-v1

return(tab)

}

MajorGroupLevel<-as.character(colDta[,MajorGroupLevel])

CompareGroupLevel<-as.character(colDta[,CompareGroupLevel])

gtab<-data.frame(Samples=colDta[,SampleCol],Group=paste0(MajorGroupLevel,connector,CompareGroupLevel))

dds<-DESeqDataSetFromMatrix(countData = countData[,gtab$Samples],colData = gtab,design = ~Group)

dds<-DESeq(dds)

res<-vector("list")

for(g in unique(MajorGroupLevel)){

###计算各组差异表格并储存

cat("###########")

cat("\n")

cat(paste0(" ",g," "))

T1vsT0<-paste0(T1,".VS.",T0)

cat(T1vsT0)

cat("\n")

T1vsT0_tab<-as.data.frame(results(dds,contrast = c("Group",paste0(g,connector,T1),paste0(g,connector,T0))))

res[[g]][["rawtab"]][[T1vsT0]]<-T1vsT0_tab

T1vsT0_tab<-ModiTab(T1vsT0_tab)

res[[g]][["GSEA"]][[T1vsT0]]<-Diff2GSEAandPlots(difftab = T1vsT0_tab,

ImmuneFunctionSets = ImmuneFunctionSets,

pCol = 6,

type = gseaplotType,

ntop = ntop,

maxoverlap = GSEAmaxoverlap,

sig = sig,RefSet = RefSet)

T2AvsT1<-paste0(T2A,".VS.",T1)

cat(T2AvsT1)

cat("\n")

T2AvsT1_tab<-as.data.frame(results(dds,contrast = c("Group",paste0(g,connector,T2A),paste0(g,connector,T1))))

res[[g]][["rawtab"]][[T2AvsT1]]<-T2AvsT1_tab

T2AvsT1_tab<-ModiTab(T2AvsT1_tab)

res[[g]][["GSEA"]][[T2AvsT1]]<-Diff2GSEAandPlots(difftab = T2AvsT1_tab,

ImmuneFunctionSets = ImmuneFunctionSets,

pCol = 6,

type = gseaplotType,

ntop = ntop,

maxoverlap = GSEAmaxoverlap,

sig = sig,RefSet = RefSet)

T2BvsT1<-paste0(T2B,".VS.",T1)

cat(T2BvsT1)

cat("\n")

T2BvsT1_tab<-as.data.frame(results(dds,contrast = c("Group",paste0(g,connector,T2B),paste0(g,connector,T1))))

res[[g]][["rawtab"]][[T2BvsT1]]<-T2BvsT1_tab

T2BvsT1_tab<-ModiTab(T2BvsT1_tab)

res[[g]][["GSEA"]][[T2BvsT1]]<-Diff2GSEAandPlots(difftab = T2BvsT1_tab,

ImmuneFunctionSets = ImmuneFunctionSets,

pCol = 6,

type = gseaplotType,

ntop = ntop,

maxoverlap = GSEAmaxoverlap,

sig = sig,RefSet = RefSet)

T2AvsT2B<-paste0(T2A,".VS.",T2B)

cat(T2AvsT2B)

cat("\n")

T2AvsT2B_tab<-as.data.frame(results(dds,contrast = c("Group",paste0(g,connector,T2A),paste0(g,connector,T2B))))

res[[g]][["rawtab"]][[T2AvsT2B]]<-T2AvsT2B_tab

T2AvsT2B_tab<-ModiTab(T2AvsT2B_tab)

res[[g]][["GSEA"]][[T2AvsT2B]]<-Diff2GSEAandPlots(difftab = T2AvsT2B_tab,

ImmuneFunctionSets = ImmuneFunctionSets,

pCol = 6,

type = gseaplotType,

ntop = ntop,

maxoverlap = GSEAmaxoverlap,

sig = sig,RefSet = RefSet)

###Merge Table

mergedTab_all<-data.frame(Genes=rownames(T2AvsT2B_tab),

log2Fc_T1vsT0=T1vsT0_tab$log2FoldChange,

log2Fc_T2AvsT1=T2AvsT1_tab$log2FoldChange,

log2Fc_T2BvsT1=T2BvsT1_tab$log2FoldChange,

log2Fc_T2AB=T2AvsT2B_tab$log2FoldChange,

pvalue_T1vsT0=T1vsT0_tab$pvalue,

pvalue_T2AvsT1=T2AvsT1_tab$pvalue,

pvalue_T2BvsT1=T2BvsT1_tab$pvalue,

pvalue_T2AB=T2AvsT2B_tab$pvalue,

padj_T1vsT0=T1vsT0_tab$padj,

padj_T2AvsT1=T2AvsT1_tab$padj,

padj_T2BvsT1=T2BvsT1_tab$padj,

padj_T2AB=T2AvsT2B_tab$padj)

rownames(mergedTab_all)<-rownames(T2AvsT2B_tab)

mergedTab_all_sig<-mergedTab_all[,2:5]

mergedTab_all_sig<-mergedTab_all_sig[which(rowSums(mergedTab_all_sig) !=0),]

res[[g]][["mergedTab_all"]]<-mergedTab_all

res[[g]][["mergedTab_all_sig"]]<-mergedTab_all_sig

}

return(res)

}
