# Supplementary material for: Multiomics profiles of genome-wide alterations in H3K27ac in different lung lobes after acute graft-versus-host disease with MSCs treatment
Source: Front Immunol. 2025 May 15;16:1570916. doi: 10.3389/fimmu.2025.1570916 (PMC12119469; doi:10.3389/fimmu.2025.1570916)
Supplement: Supplementary file 1 [file DataSheet1.zip › Figure4.Function/ExtractGSEAres.docx]

ExtractGSEAres<-function(lst,geneset,gtabForHeatmap,ptype="pvalue"){

###

### ptype: pvalue,qvalue,padj

require(rlist)

OutRes<-vector("list")

L1<-names(lst)

for(l in L1){

L1lst<-lst[[l]][["GSEA"]]

L2<-names(L1lst)

for(i in L2){

print(paste0(l,"________________",i))

if(class(L1lst[[i]][["RawRes"]][[geneset]])=="try-error"){

next

}else{

L2tab<-L1lst[[i]][["RawRes"]][[geneset]]@result[,c(1,4,5,6,7,8)]

L2tab<-data.frame(Group1=l,Group2=i,L2tab)

OutRes[[paste0(l,"_",i)]]<-L2tab

}

}

}

ggplotTab<-list.rbind(OutRes)

ggplotTab$Groups<-paste0(ggplotTab$Group1,"_",ggplotTab$Group2)

clons<-unique(ggplotTab$Groups)

rowns<-unique(ggplotTab$ID)

heatTab<-matrix(data = 0,

nrow = length(rowns),

ncol = length(clons))

colnames(heatTab)<-clons

rownames(heatTab)<-rowns

ptab<-heatTab

qtab<-heatTab

adjpTab<-heatTab

for(i in clons){

nes<-OutRes[[i]]

for(j in rowns){

if(j %in% unique(nes$ID)){

NES<-nes$NES[which(nes$ID == j)]

pv<-nes$pvalue[which(nes$ID == j)]

qv<-nes$qvalue[which(nes$ID == j)]

padj<-nes$p.adjust[which(nes$ID == j)]

}else{

NES<-0

pv<-1

qv<-1

padj<-1

}

heatTab[j,i]<-NES

ptab[j,i]<-pv

qtab[j,i]<-qv

adjpTab[j,i]<-padj

}

}

require(pheatmap)

if(ptype == "pvalue"){pmat<-ptab}

if(ptype == "qvalue"){pmat<-qtab}

if(ptype == "padj"){pmat<-adjpTab}

return(list(ggplotTab=ggplotTab,heatTabs=list(heatTab=heatTab,ptab=ptab,qtab=qtab,adjpTab=adjpTab)))

}
