# Supplementary material for: Multiomics profiles of genome-wide alterations in H3K27ac in different lung lobes after acute graft-versus-host disease with MSCs treatment
Source: Front Immunol. 2025 May 15;16:1570916. doi: 10.3389/fimmu.2025.1570916 (PMC12119469; doi:10.3389/fimmu.2025.1570916)
Supplement: Supplementary file 1 [file DataSheet1.zip › Figure4.Function/extractSpecificGenesFromLst.docx]

extractSpecificGenesFromLst<-function(lst,tarName="L_MvG_up"){

lst1<-lst[[tarName]]

lst[[tarName]]<-NULL

for(i in names(lst)){

lst1<-lst1[which(! lst1 %in% lst[[i]])]

}

return(lst1)

}
