# Supplementary material for: Multiomics profiles of genome-wide alterations in H3K27ac in different lung lobes after acute graft-versus-host disease with MSCs treatment
Source: Front Immunol. 2025 May 15;16:1570916. doi: 10.3389/fimmu.2025.1570916 (PMC12119469; doi:10.3389/fimmu.2025.1570916)
Supplement: Supplementary file 1 [file DataSheet1.zip › Figure4.Function/GenesLst2Upset.docx]

GenesLst2Upset<-function(lst,

nsets = 100, # 绘制的最大集合个数

nintersects = 30, #绘制的最大交集个数，NA则全部绘制

order.by = "freq", # 矩阵中的交点是如何排列的。 "freq"根据交集个数排序，"degree"根据

keep.order = T, # 保持设置与使用sets参数输入的顺序一致。默认值是FALSE，它根据集合的大小排序。

mb.ratio = c(0.6,0.4), # 左侧和上方条形图的比例关系

text.scale = 1 # 文字标签的大小

){

require(UpSetR)

p1<-upset(fromList(lst), # fromList一个函数，用于将列表转换为与UpSetR兼容的数据形式。

nsets = nsets, # 绘制的最大集合个数

nintersects = nintersects, #绘制的最大交集个数，NA则全部绘制

order.by = order.by, # 矩阵中的交点是如何排列的。 "freq"根据交集个数排序，"degree"根据

keep.order = keep.order, # 保持设置与使用sets参数输入的顺序一致。默认值是FALSE，它根据集合的大小排序。

mb.ratio = mb.ratio, # 左侧和上方条形图的比例关系

text.scale = text.scale # 文字标签的大小

)

return(p1)

}
