# Supplementary material for: Multiomics profiles of genome-wide alterations in H3K27ac in different lung lobes after acute graft-versus-host disease with MSCs treatment
Source: Front Immunol. 2025 May 15;16:1570916. doi: 10.3389/fimmu.2025.1570916 (PMC12119469; doi:10.3389/fimmu.2025.1570916)
Supplement: Supplementary file 1 [file DataSheet1.zip › Figure4.Function/ppheatmap.docx]

ppheatmap<-function(dat,pmat,

annoRowtab=NULL,annoColtab=NULL,

psig=0.05,corsig=0.3,

cluster_rows=T,cluster_cols=F,rmStr="HALLMARK_"){

idenFun<-function(x) return(all(x>0.05))

rns<-rownames(dat)

rns<-gsub(rmStr,"",rns)

rownames(dat)<-rns

colnames(pmat)<-colnames(dat)

rownames(pmat)<-rns

keptRow<-which(!apply(pmat,1,idenFun))

pmat<-pmat[keptRow,]

dat<-dat[keptRow,]

disMatrix<-as.matrix(ifelse(pmat<psig & abs(dat)>corsig,"*",""),nrow(pmat))

p1<-pheatmap(dat,display_numbers = disMatrix,

annotation_row = annoRowtab,

annotation_col = annoColtab,

cluster_rows = cluster_rows,

cluster_cols = cluster_cols)

return(p1)

}
