# Supplementary material for: Multiomics profiles of genome-wide alterations in H3K27ac in different lung lobes after acute graft-versus-host disease with MSCs treatment
Source: Front Immunol. 2025 May 15;16:1570916. doi: 10.3389/fimmu.2025.1570916 (PMC12119469; doi:10.3389/fimmu.2025.1570916)
Supplement: Supplementary file 3 [file DataSheet3.zip › Figure 4.Codes/Figure.4A codes.docx]

##重点关注promoter区域的基因

wgyPeakPromoterDiff1<-ExtractAbsDiff_withoutpadj(wgyMasterPeakCount1_annotated_promoter_forGSVA,

colDta = groupfile,

SampleCol = 1,

MajorGroupLevel = 3,

CompareGroupLevel = 2

)

wgyPeakPromoterDiff1_HALLMARK<-ExtractGSEAres(wgyPeakPromoterDiff1,"HallMarker",gtabForHeatmap = gtabForHeatmap)

###Figure 4A

wgyPeakPromoterDiff1_HALLMARK_pheat<-ppheatmap(wgyPeakPromoterDiff1_HALLMARK$heatTabs$heatTab,pmat = wgyPeakPromoterDiff1_HALLMARK$heatTabs$ptab)
