# Supplementary material for: Multiomics profiles of genome-wide alterations in H3K27ac in different lung lobes after acute graft-versus-host disease with MSCs treatment
Source: Front Immunol. 2025 May 15;16:1570916. doi: 10.3389/fimmu.2025.1570916 (PMC12119469; doi:10.3389/fimmu.2025.1570916)
Supplement: Supplementary file 3 [file DataSheet3.zip › Figure 4.Codes/Figure.4B codes.docx]

wgyPeakPromoterDiff_UpDownLst<-DiffTablst2Upset(wgyPeakPromoterDiff)

library(UpSetR)

##Figure 4B

wgyPeakPromoterDiff_UpDownLst_upset<-GenesLst2Upset(wgyPeakPromoterDiff_UpDownLst,

mb.ratio = c(0.3,0.7),

nintersects = 20)
