# Supplementary material for: Multiomics profiles of genome-wide alterations in H3K27ac in different lung lobes after acute graft-versus-host disease with MSCs treatment
Source: Front Immunol. 2025 May 15;16:1570916. doi: 10.3389/fimmu.2025.1570916 (PMC12119469; doi:10.3389/fimmu.2025.1570916)
Supplement: Supplementary file 3 [file DataSheet3.zip › Figure 4.Codes/Figure.4C codes.docx]

Figure4C.lst<-list()

Figure4C.lst$L.up<-extractSpecificGenesFromLst(wgyPeakPromoterDiff_UpDownLst,tarName = "L_MvG_up")

Figure4C.lst$L.down<-extractSpecificGenesFromLst(wgyPeakPromoterDiff_UpDownLst,tarName = "L_MvG_down")

Figure4C.lst$R3.up<-extractSpecificGenesFromLst(wgyPeakPromoterDiff_UpDownLst,tarName = "R3_MvG_up")

Figure4C.lst$L.up.peak<-subset(wgyMasterPeakCount1_annotated_forShiftDetect,geneId %in% Figure4C.lst$L.up & annotation == "Promoter")$ID

Figure4C.lst$L.up.peakTab<-subset(wgyMasterPeak_tab,Genes %in% Figure4C.lst$L.up.peak)

write.table(Figure4C.lst$L.up.peakTab,file = "L.up.peak.bed",quote = F,sep = "\t",row.names = F,col.names = F)

Figure4C.lst$L.down.peak<-subset(wgyMasterPeakCount1_annotated_forShiftDetect,geneId %in% Figure4C.lst$L.down & annotation == "Promoter")$ID

Figure4C.lst$L.down.peakTab<-subset(wgyMasterPeak_tab,Genes %in% Figure4C.lst$L.down.peak)

write.table(Figure4C.lst$L.down.peakTab,file = "L.down.peak.bed",quote = F,sep = "\t",row.names = F,col.names = F)

Figure4C.lst$R3.up.peak<-subset(wgyMasterPeakCount1_annotated_forShiftDetect,geneId %in% Figure4C.lst$R3.up & annotation == "Promoter")$ID

Figure4C.lst$R3.up.peakTab<-subset(wgyMasterPeak_tab,Genes %in% Figure4C.lst$R3.up.peak)

write.table(Figure4C.lst$R3.up.peakTab,file = "R3.up.peak.bed",quote = F,sep = "\t",row.names = F,col.names = F)

library(ChIPseeker)

Figure4C.peaksForPlot<-GenomicRanges::GRangesList(L.up=readPeakFile("L.up.peak.bed"),

L.down=readPeakFile("L.down.peak.bed"),

R3.up=readPeakFile("R3.up.peak.bed"))

###Figure 4C

covplot(Figure4C.peaksForPlot)+facet_grid(chr ~ .id)
