# Supplementary figures and images for: Multiomics profiles of genome-wide alterations in H3K27ac in different lung lobes after acute graft-versus-host disease with MSCs treatment
Source: Front Immunol. 2025 May 15;16:1570916. doi: 10.3389/fimmu.2025.1570916 (PMC12119469; doi:10.3389/fimmu.2025.1570916)

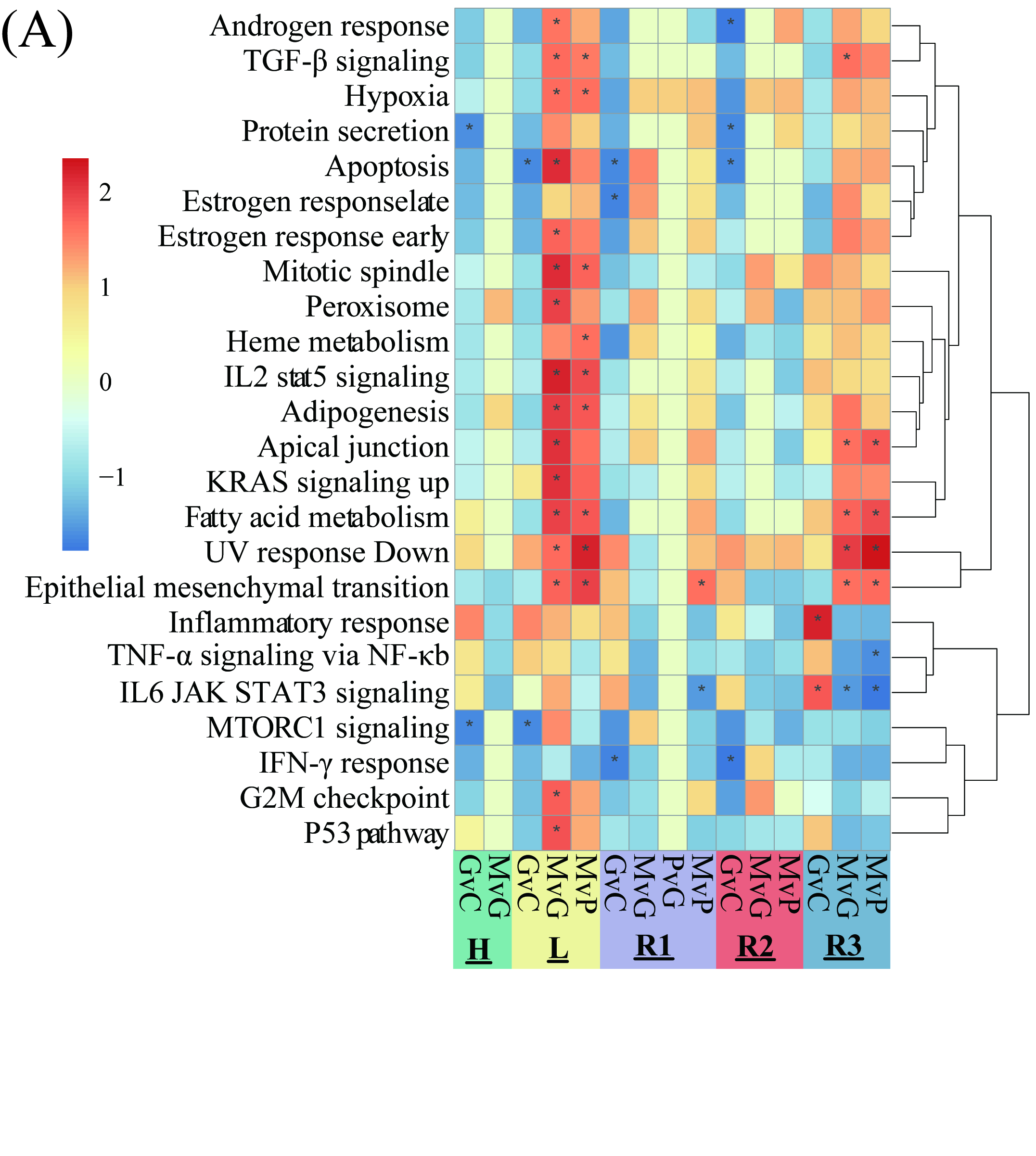

Supplement: Supplementary file 3 [file DataSheet3.zip › Figure 4.Picture/Figure.4A.tif]

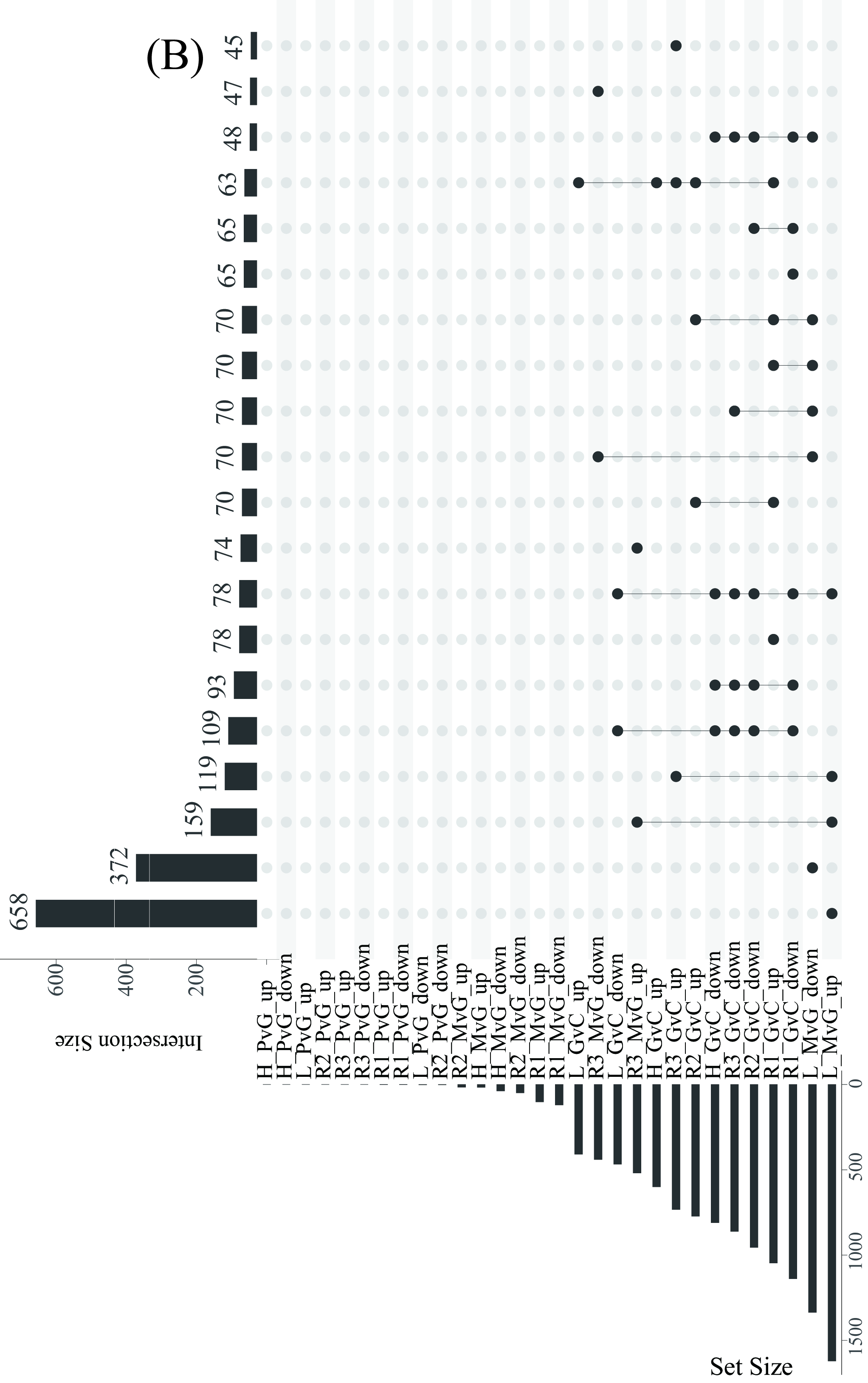

Supplement: Supplementary file 3 [file DataSheet3.zip › Figure 4.Picture/Figure.4B.tif]

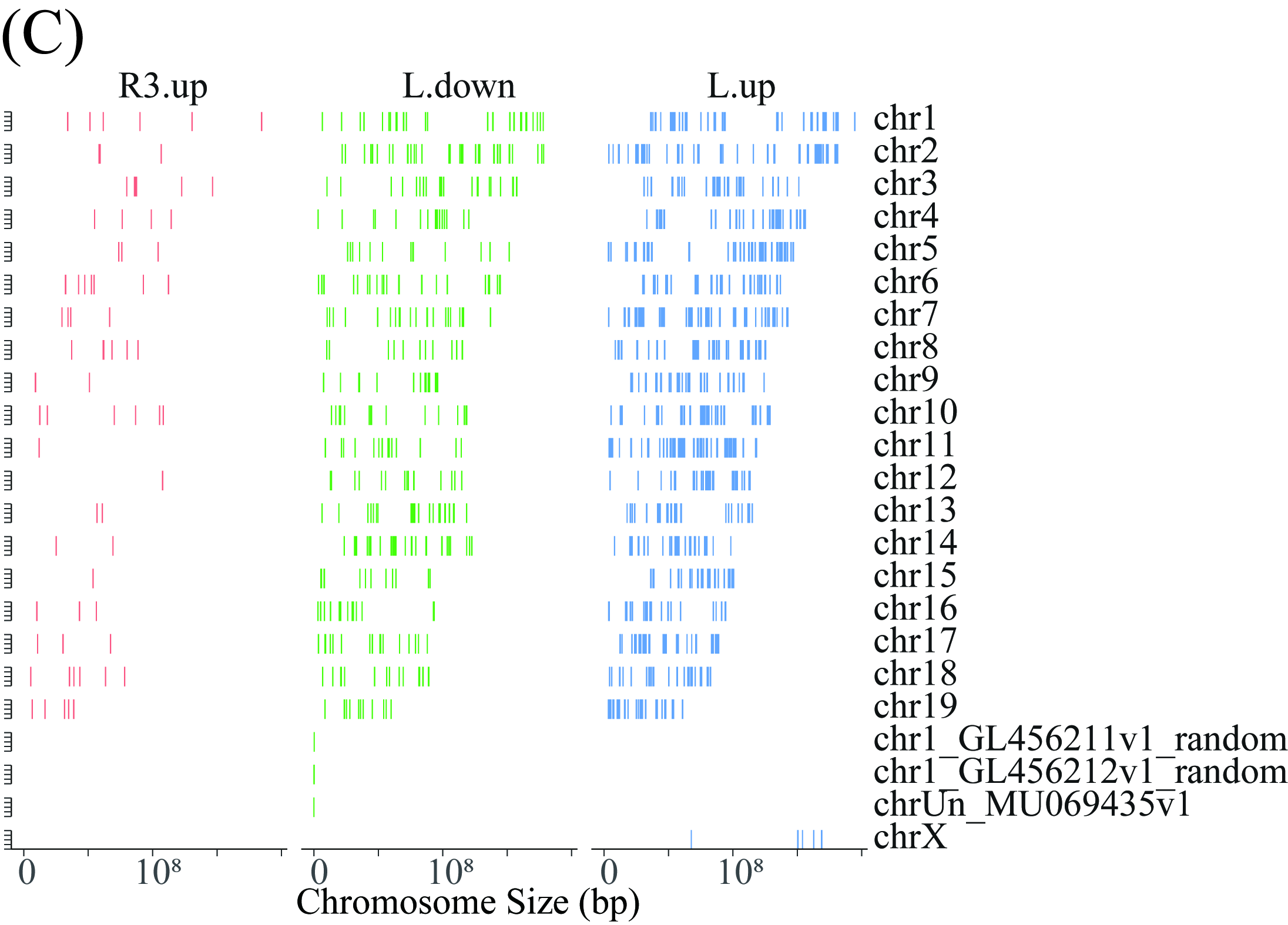

Supplement: Supplementary file 3 [file DataSheet3.zip › Figure 4.Picture/Figure.4C.tif]

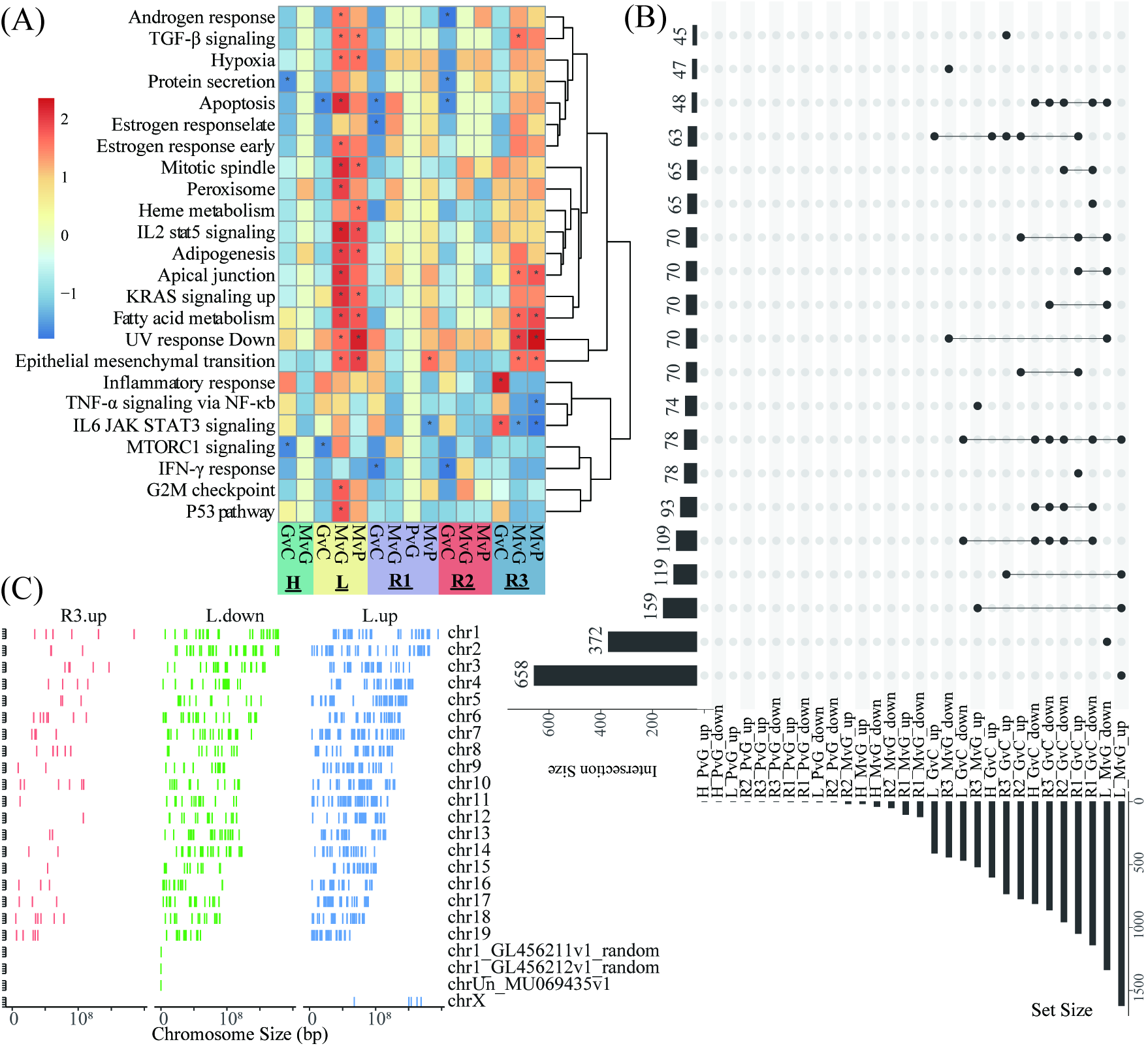

Supplement: Supplementary file 3 [file DataSheet3.zip › Figure 4.Picture/Figure4.tif]
