# Supplementary material for: Multiomics profiles of genome-wide alterations in H3K27ac in different lung lobes after acute graft-versus-host disease with MSCs treatment
Source: Front Immunol. 2025 May 15;16:1570916. doi: 10.3389/fimmu.2025.1570916 (PMC12119469; doi:10.3389/fimmu.2025.1570916)
Supplement: Supplementary file 5 [file Table1.docx]

Supplementary Figures


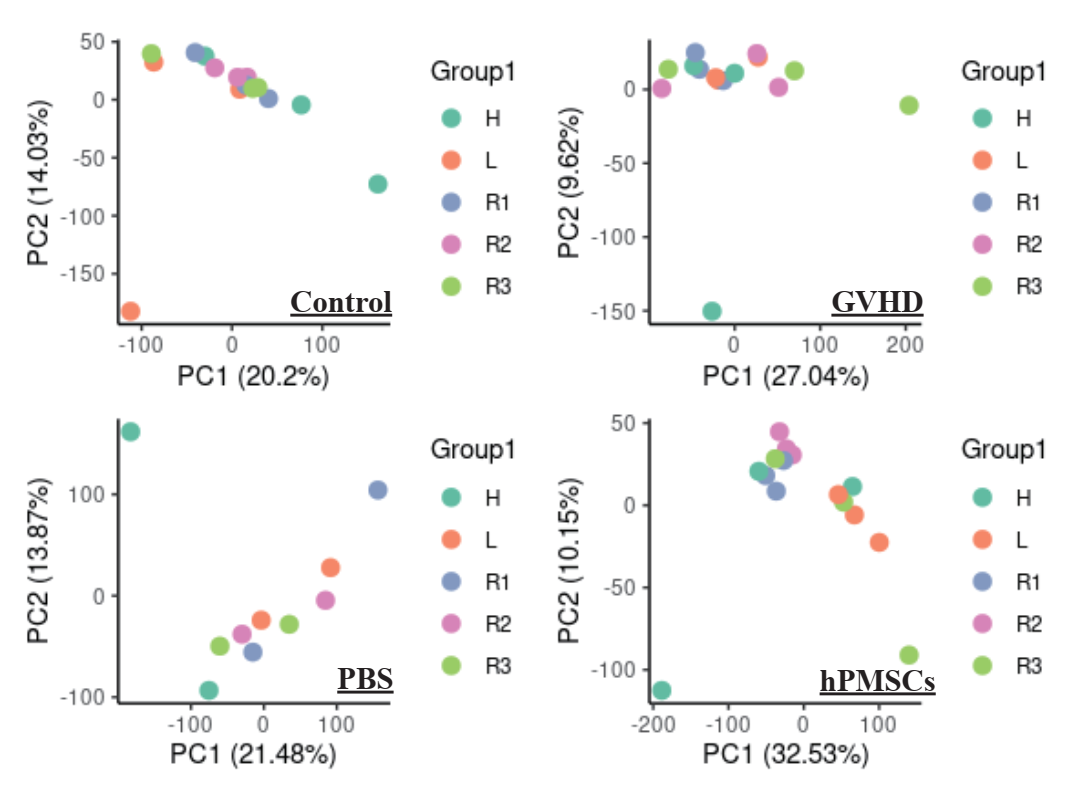


Figure S1. Principal component analysis of the transcriptome in different groups.


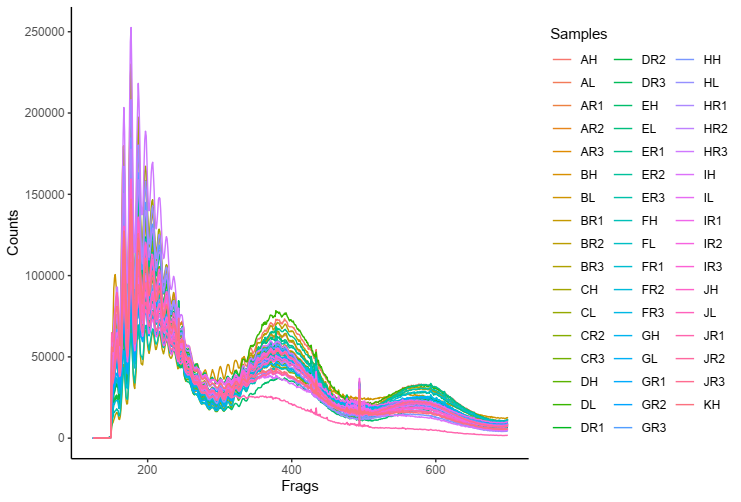


Figure S2. Distribution of mapped fragment size


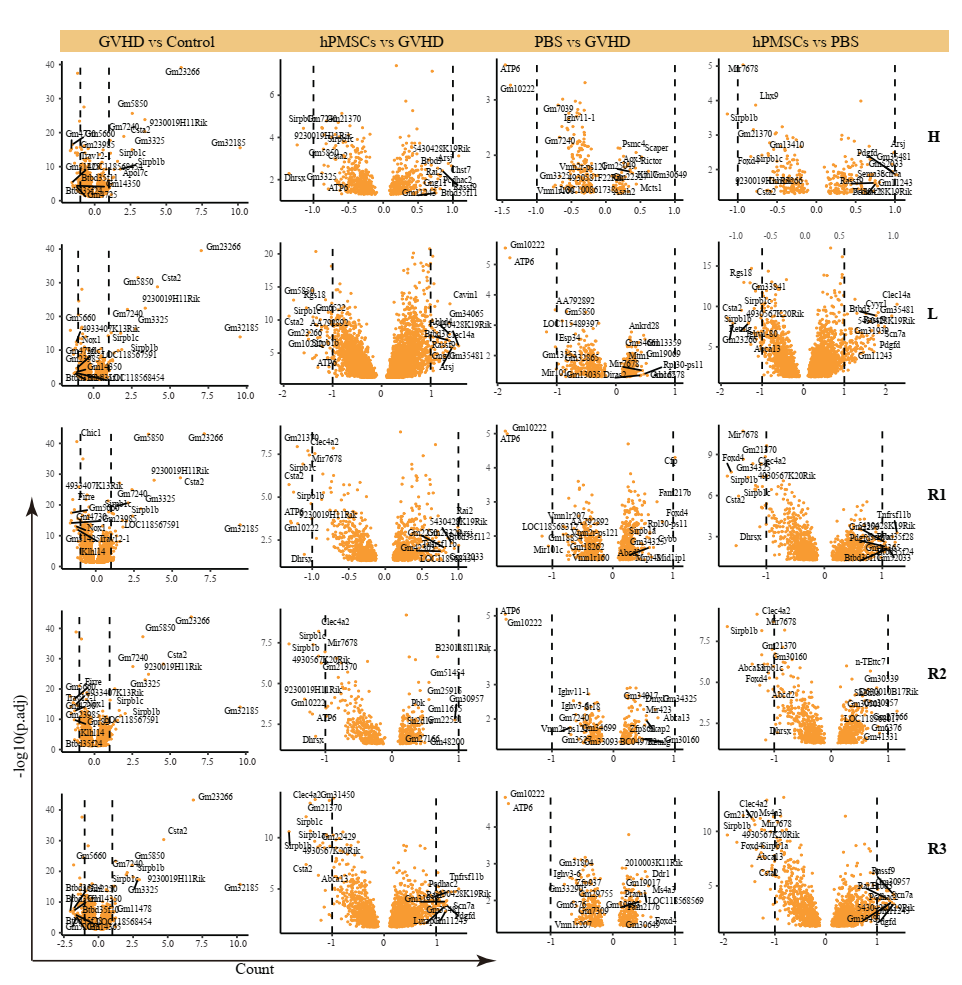


Figure S3: Volcano plot illustrating genes with significantly different number of reads in promoter regions in all comparisons of all lobes


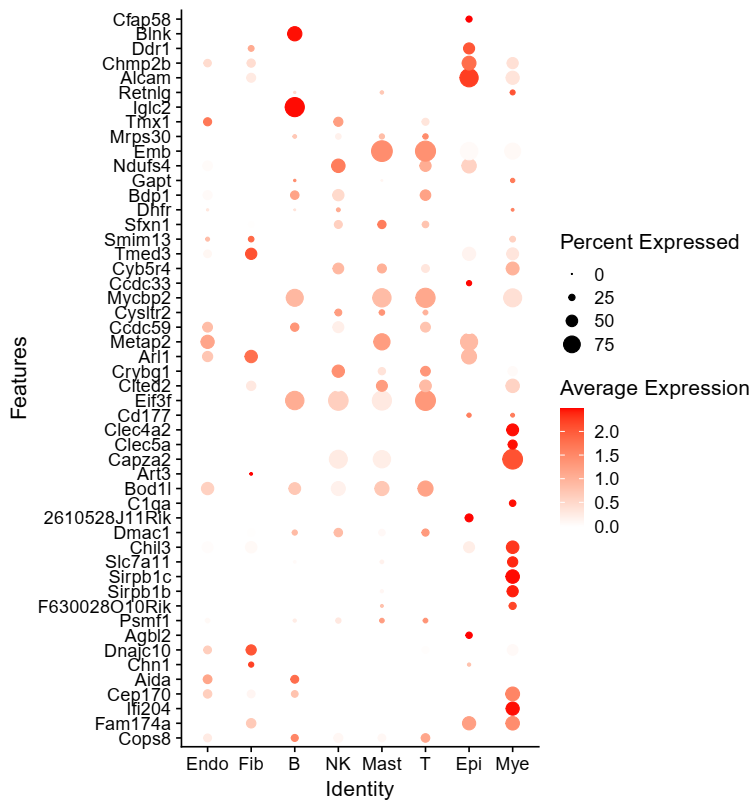


Figure S4. Dotplot illustrating the a-DEGs of MvG in L lobe. Only the genes with average expression > 0.1 were shown here.


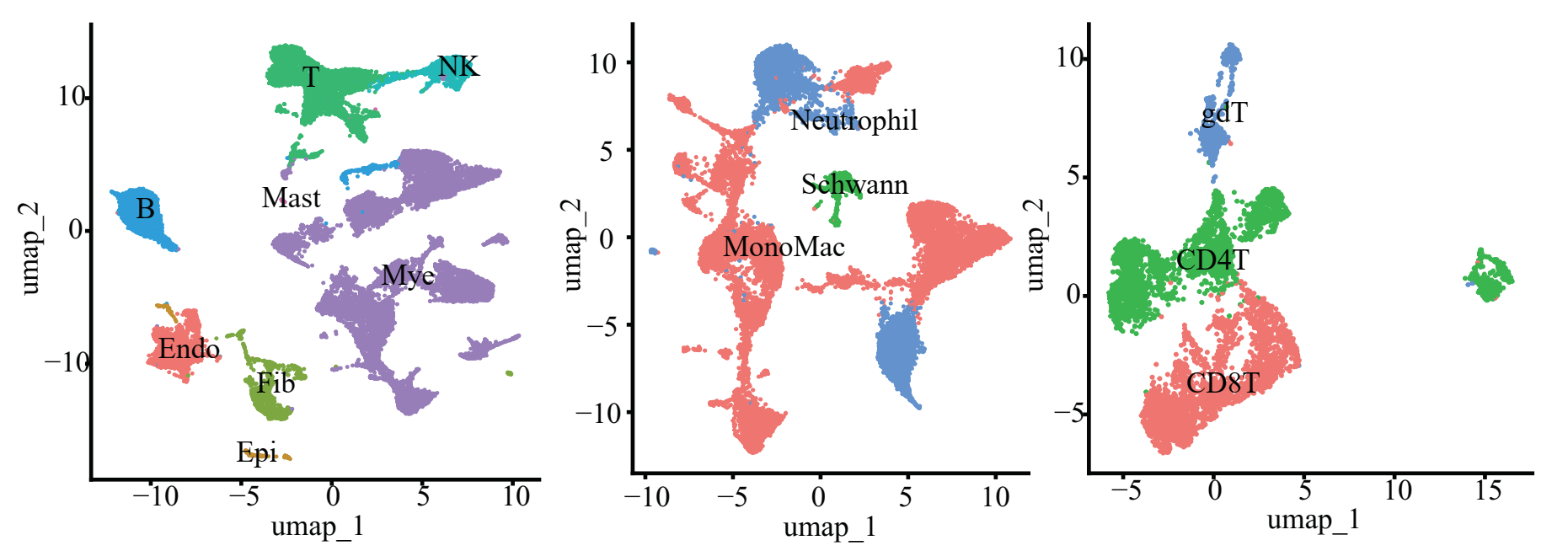


Figure S5. UMAP plot showing the cell types of all cells, and the subsets of Myeloid and the T cells.
